# Reduced QED with Few Planes and Fermion Gap Generation

**DOI:** 10.3390/e25091317

**Published:** 2023-09-09

**Authors:** Eduard V. Gorbar, Valery P. Gusynin, Maxim R. Parymuda

**Affiliations:** 1 Department of Physics, Taras Shevchenko National Kyiv University, 03022 Kyiv, Ukraine; gorbar@knu.ua (E.V.G.); parimuda2016@gmail.com (M.R.P.); 2 Bogolyubov Institute for Theoretical Physics, 03143 Kyiv, Ukraine

**Keywords:** reduced QED, effective action, gap generation

## Abstract

The formalism of reduced quantum electrodynamics is generalized to the case of heterostructures composed of a few atomically thick layers, and the corresponding effective (2+1)-dimensional gauge theory is formulated. This dimensionally reduced theory describes charged fermions confined to *N* planes and contains *N* vector fields with Maxwell’s action modified by non-local form factors whose explicit form is determined. Taking into account the polarization function, the explicit formulae for the screened electromagnetic interaction are presented in the case of two and three layers. For a heterostructure with two atomically thick layers and charged fermions described by the massless Dirac equation, the dynamical gap generation of the excitonic type is studied. It is found that additional screening due to the second layer increases the value of the critical coupling constant for the gap generation compared to that in graphene.

## 1. Introduction

There are many physical systems where charged fermions are confined to geometric structures with spatial dimensions less than three. Quantum dots, quantum wires, and atomically thick planar systems provide the most familiar examples, where, unlike the charged fermions, the electromagnetic field propagates beyond the confining geometries. Such systems are described by the usual 3D Maxwell equations with sources localized in dimensions less than three. To efficiently describe such physical systems, the formalism of reduced quantum electrodynamics (reduced QED) [1] or, equivalently, pseudo-quantum electrodynamics (PQED) [2], was developed (for earlier studies, see also [3,4]). A more general model of reduced QED with fermions living in $d_{e}$-dimensional spacetime interacting via the exchange of massless bosons in $d_{\gamma }$ dimensions ($d_{e}<d_{\gamma }$), called mixed-dimensional QED, was proposed in Ref. [5].

It is also worth mentioning that the idea of matter living in fewer spatial dimensions than the force carrier was considered in the theory of gravity as well, where it is known as the braneworld [6,7]. In braneworld models, it is assumed that our visible three-dimensional universe is restricted to a brane inside a higher-dimensional space. This assumption could naturally explain the weakness of gravity relative to other fundamental forces. Indeed, unlike the electromagnetic, weak, and strong nuclear forces localized on the brane, gravity propagates in the ambient higher-dimensional spacetime that results in a much weaker gravitational attraction compared to the other fundamental forces.

The motivation for the formulation of reduced QED is quite straightforward. Since charged fermions are localized in subspaces of lower dimensions, it is natural and, in addition, more convenient to describe their interaction by means of an effective dimensionally reduced gauge theory. Reduced QED could be used to study graphene [8], surface states in topological insulators [9], artificial graphene-like systems [10], etc. It was shown that reduced QED, despite being non-local, is unitary [11]. Supplanting it with fermion mass term, reduced QED could be used to describe the exciton spectrum in transition metal dichalcogenide monolayers [12] and the renormalization of their band gap [13] induced by interactions.

The dynamical mass generation in reduced QED, taking into account the screening effects, was studied in [1]. The analysis of the Schwinger–Dyson equations revealed rich and quite nontrivial dynamics in which the conformal symmetry and its breakdown play a crucial role. Reduced QED with one plane is conformally invariant because the original (3+1)-dimensional QED with massless fermions is conformally invariant and the vacuum polarization function for massless fermions in (1+1) and (2+1) dimensions is conformally invariant as well. Conformal aspects of reduced QED were highlighted in [14,15]. The analysis of dynamical mass generation in reduced QED [1] was extended to the study of the excitonic-type gap generation in graphene [8,16,17,18,19,20,21], followed by lattice simulations [22,23].

The possibility to generate in a controlled way a fermion gap in graphene and graphene-like materials, which is much needed for the development of graphene-based transistors, motivated further studies of reduced QED. For this, a detailed analysis of the gap generation in reduced QED was carried out, taking into account the dynamical screening and the wave function renormalization in the two-loop approximation [24,25]. It was also shown that additional four-fermion interactions diminish the value of the critical coupling constant [26], similar to the case of monolayer graphene [18]. A review of the electron–electron interaction effects in low-dimensional Dirac materials employing the reduced QED formalism was given in [27].

In addition to monolayer materials, multilayer nanostructures are also being actively studied in condensed matter physics. It is fair to say that the experimental discovery of graphene [28] and other two-dimensional (2D) crystals [29] led to a revolution in the study of layered nanomaterials. Using atomically thick materials such as hexagonal boron nitride, chalcogenides, black phosphorus, etc., the van der Waals assembly provided a practical way to combine 2D crystals in heterostructures with designer functional possibilities [30].

Two-layer materials are the simplest multilayer heterostructures. It was shown that double-layer Dirac systems composed of two graphene layers separated by a thin dielectric layer and charged oppositely provide one of the most realistic physical systems to achieve the exciton condensation because the electron and hole Fermi surfaces in two layers are perfectly nested in this case [31,32,33,34,35]. It was found that the dynamical screening of the Coulomb interaction plays an essential role in determining the properties of the exciton condensate in double-layer Dirac systems [36], and even with the screening effects taken into account, the excitonic gap can reach values of the order of the Fermi energy.

In view of the active study of multilayer nanostructures, we aim in this paper to extend the formalism of reduced QED to the case of heterostructures composed of *N* layers. To demonstrate the usefulness of the obtained extension, we study, taking into account the screening effects, the gap generation for massless Dirac fermions confined to two equivalent planes.

The paper is organized as follows. The effective reduced theory for fermions confined to *N* planes is derived in Section 2. The screening effects due to massless fermions in a heterostructure with *N* equivalent planes are considered in Section 3. The fermion gap equation is derived in Section 4. The solutions of the gap equation are found and the critical coupling constant is determined in Section 5. The obtained results are summarized in Section 6.

## 2. Reduced QED for Heterostructure with *N* Planes

Let us find an effective action for charged particles confined to *N* two-dimensional planes. In Euclidean space, the electrodynamic action of the corresponding system is given by

(1) $$S=\int d^{4}x\;\left(\frac{1}{4}F_{\mu \nu }^{2}+A_{\mu }j^{\mu }+\frac{1}{2\xi }{\left(\partial_{\mu }A^{\mu }\right)}^{2}\right),$$

where $F_{\mu \nu }=\partial_{\mu }A_{\nu }-\partial_{\nu }A_{\mu }$ is the electromagnetic field strength tensor, $j^{\mu }$ is the electric current of charged particles confined to *N* planes, and ξ is the gauge fixing parameter. In the case of equidistantly separated planes in the *z*-direction with the distance *a* between the planes, the electric current is given by

(2) $$j^{\mu }\left(x\right)=\left\{\begin{array}{cc} \sum_{n=1}^{N}j_{n}^{\mu }\left(x^{0},x\right)\delta \left(z-na\right) & \mathrm{for}\;\mu =0,1,2,\\ 0 & \mathrm{for}\;\mu =3, \end{array}\right.$$

where x $=(x_{1},x_{2})$ is a two-dimensional vector in the planes and the delta-function δ(z−na) appears because charged particles are confined to the corresponding planes. Integrating over the electromagnetic field $A_{\mu }$ in the functional integral, we can easily obtain the interaction term of the action for charged particles

(3) $$S=\frac{1}{2}\int d^{4}xd^{4}y\;j^{\mu }\left(x\right)D_{\mu \nu }\left(x-y\right)j^{\nu }\left(y\right),$$

where $D_{\mu \nu }\left(x-y\right)=$$\frac{1}{-□}\left(\delta_{\mu \nu }-\left(1-\xi \right)\frac{\partial_{\mu }\partial_{\nu }}{□}\right)$ $\delta^{4}\left(x-y\right)$ is the photon propagator and $□=\partial_{\mu }^{2}$. Substituting the expression for the current (2), we obtain

(4) $$S=\frac{1}{2}\sum_{n,m=0}^{N}\int d^{3}xd^{3}y\;j_{n}^{\mu }\left(x\right)D_{\mu \nu }\left(x-y,\left(n-m\right)a\right)j_{m}^{\nu }\left(y\right),$$

where, now, indices μ,ν run over the values 0,1,2 and $x=(x_{0},x_{1},x_{2})$, $y=(y_{0},y_{1},y_{2})$. In momentum space, we have, for the reduced photon propagator

(5) $$\begin{array}{cc} \; & D_{\mu \nu }^{nm}\left(x-y\right)\equiv D_{\mu \nu }\left(x-y,\left(n-m\right)a\right)=\\ \; & =\int \frac{d^{3}kdk_{3}}{{\left(2\pi \right)}^{4}}\;e^{ik\left(x-y\right)+ik_{3}a\left(n-m\right)}\left(\delta_{\mu \nu }-\left(1-\xi \right)\frac{k_{\mu }k_{\nu }}{k_{3}^{2}+k^{2}}\right)\frac{1}{k_{3}^{2}+k^{2}}\\ \; & =\int \frac{d^{3}k}{{\left(2\pi \right)}^{3}}\;e^{ik(x-y)}D_{\mu \nu }^{nm}\left(k\right), \end{array}$$

where

(6) $$D_{\mu \nu }^{nm}\left(k\right)=\frac{e^{-|n-m|ak}}{2k}\left[\delta_{\mu \nu }-\left(1-\xi \right)\frac{k_{\mu }k_{\nu }}{2k^{2}}\left(|n-m|ak+1\right)\right],\;n,m=1,...,N,\;k=\left|k\right|.$$

To obtain the reduced QED theory for the general case of *N* planes, which reproduces upon the functional integration on gauge fields the interaction term (3) for charged particles, it is useful to begin with the study of a heterostructure composed of two planes.

### 2.1. Two Planes

For charges in the same plane, n=m, i.e., $x_{3}=y_{3}$, Equation (6) defines the following effective interaction in a configuration space in each of the two planes:

(7) $$D_{\mu \nu }^{11}=D_{\mu \nu }^{22}=\frac{1}{2\sqrt{-□}}\left[\delta_{\mu \nu }-\left(1-\xi \right)\frac{\partial_{\mu }\partial_{\nu }}{2□}\right]\delta^{3}\left(x-y\right),\;\mu ,\nu \neq 3,$$

which, of course, coincides exactly with that in the reduced QED with one plane [1]. For interacting charges situated in two different planes separated by distance *a*, we find the effective interaction:

(8) $$D_{\mu \nu }^{12}=D_{\mu \nu }^{21}=\frac{e^{-a\sqrt{-□}}}{2\sqrt{-□}}\left[\delta_{\mu \nu }-\left(1-\xi \right)\frac{\partial_{\mu }\partial_{\nu }}{2□}\left(a\sqrt{-□}+1\right)\right]\delta^{3}\left(x-y\right).$$

Thus, we obtain the following reduced (2+1)-dimensional action:

(9) $$S_{\mathrm{int}}=\frac{1}{2}\int d^{3}xd^{3}y\;j^{\mu }\left(x\right){\hat{D}}_{\mu \nu }\left(x-y\right)j^{\nu }\left(y\right),$$

where $j^{\mu }={\left(j_{1}^{\mu },j_{2}^{\mu }\right)}^{T}$ are the electric currents in the planes and

(10) $${\hat{D}}_{\mu \nu }=\left(\begin{array}{cc} D_{\mu \nu }^{11} & D_{\mu \nu }^{12}\\ D_{\mu \nu }^{21} & D_{\mu \nu }^{22} \end{array}\right).$$

Clearly, to obtain the interaction action (9) in an (2+1)-dimensional effective electrodynamic action, we should introduce two auxiliary vector fields: $A_{\mu }^{1}$ and $A_{\mu }^{2}$. It is convenient to use the Feynman gauge ξ=1 because the elements $D_{\mu \nu }^{11}$ and $D_{\mu \nu }^{12}$ in Equations (7) and (8) have the same tensor structure in this gauge. Then, a general effective (2+1)-dimensional action for charges confined to two planes interacting with two vector fields $A_{\mu }^{1}$ and $A_{\mu }^{2}$ is given by

(11) $$\begin{array}{cc} S_{\mathrm{eff}}= & \;\int d^{3}x[\frac{1}{4}\left(\begin{array}{cc} F_{\mu \nu }^{1}, & F_{\mu \nu }^{2} \end{array}\right)\;{\left(\begin{array}{cc} X^{11} & X^{12}\\ X^{21} & X^{22} \end{array}\right)}^{\mu \nu \alpha \beta }\;\left(\begin{array}{c} F_{\alpha \beta }^{1}\\ F_{\alpha \beta }^{2} \end{array}\right)+A_{\mu }^{1}j_{1}^{\mu }+A_{\mu }^{2}j_{2}^{\mu }\\ \; & \;+\frac{1}{2}\partial_{\mu }\left(\begin{array}{cc} A_{\mu }^{1}, & A_{\mu }^{2} \end{array}\right)\left(\begin{array}{cc} Y^{11} & Y^{12}\\ Y^{21} & Y^{22} \end{array}\right)\partial_{\nu }\left(\begin{array}{c} A_{\nu }^{1}\\ A_{\nu }^{2} \end{array}\right)], \end{array}$$

where $\hat{Y}$ has the same form as $\hat{X}$ in the Feynman gauge, i.e., $\hat{Y}=\hat{X}$.

Integrating in the functional integral with action (11) over $A_{\mu }^{1}$ and $A_{\mu }^{2}$, we should obtain the interaction action (9). This condition results in the equation which defines $X^{\mu \nu \alpha \beta }$. In the Feynman gauge, we have

(12) $$\begin{array}{c} {\hat{D}}_{\mu \nu }^{F}=\delta_{\mu \nu }{\hat{D}}^{F},\;\;{\hat{D}}^{F}=\frac{1}{2\sqrt{-□}}\left(\begin{array}{cc} 1 & e^{-a\sqrt{-□}}\\ e^{-a\sqrt{-□}} & 1 \end{array}\right) \end{array}$$

or, in momentum space,

(13) $$\begin{array}{c} {\hat{D}}_{\mu \nu }^{F}\left(k\right)=\frac{\delta_{\mu \nu }}{2k}\left(\begin{array}{cc} 1 & e^{-ak}\\ e^{-ak} & 1 \end{array}\right)\equiv \delta_{\mu \nu }{\hat{D}}^{F}\left(k\right). \end{array}$$

Therefore, the operator $X^{\mu \nu \alpha \beta }$ has a very simple structure in indices μ,ν,α,β, i.e., $X^{\mu \nu \alpha \beta }={\hat{X}}_{2}\delta^{\mu \alpha }\delta^{\nu \beta }$, where the operator ${\hat{X}}_{2}$ is a 2 by 2 matrix with indices taking values of planes 1 and 2. Further, in order to obtain the effective interaction (9), we should find ${\hat{X}}_{2}$ by solving the operator equation:

(14) $$-□{\hat{X}}_{2}{\hat{D}}^{F}=1.$$

This results in

(15) $${\hat{X}}_{2}=\frac{2}{\sqrt{-□}\left(1-e^{-2a\sqrt{-□}}\right)}\left(\begin{array}{cc} 1 & -e^{-a\sqrt{-□}}\\ -e^{-a\sqrt{-□}} & 1 \end{array}\right)$$

or, in momentum space,

(16) $${\hat{X}}_{2}\left(k\right)=\frac{2}{k(1-e^{-2ak})}\left(\begin{array}{cc} 1 & -e^{-ak}\\ -e^{-ak} & 1 \end{array}\right).$$

Thus, the effective action for charged particles confined to two planes and interacting with two gauge fields has the following form in the Feynman gauge:

(17) $$\begin{array}{cc} & S_{eff}=\;\int d^{3}x[\frac{1}{4}\left(\begin{array}{cc} F_{\mu \nu }^{1}, & F_{\mu \nu }^{2} \end{array}\right)\;\frac{2}{\sqrt{-□}\left(1-e^{-2a\sqrt{-□}}\right)}\left(\begin{array}{cc} 1 & -e^{-a\sqrt{-□}}\\ -e^{-a\sqrt{-□}} & 1 \end{array}\right)\;\left(\begin{array}{c} F_{\mu \nu }^{1}\\ F_{\mu \nu }^{2} \end{array}\right)\;+A_{\mu }^{1}j_{\mu }^{1}\\ \; & +A_{\mu }^{2}j_{\mu }^{2}+\frac{1}{2}\partial_{\mu }\left(\begin{array}{cc} A_{\mu }^{1}, & A_{\mu }^{2} \end{array}\right)\frac{2}{\sqrt{-□}\left(1-e^{-2a\sqrt{-□}}\right)}\left(\begin{array}{cc} 1 & -e^{-a\sqrt{-□}}\\ -e^{-a\sqrt{-□}} & 1 \end{array}\right)\partial_{\nu }\left(\begin{array}{c} A_{\nu }^{1}\\ A_{\nu }^{2} \end{array}\right)]. \end{array}$$

Having solved the case of two planes, we are ready to proceed to the general case of *N* planes.

### 2.2. N Planes

As in the case of two planes considered above, the tensor structure of all elements $D_{\mu \nu }^{nm}$ is the same in the Feynman gauge ξ=1. Then, we have the following equation for ${\hat{X}}_{N}$ in momentum space:

(18) $$k^{2}{\hat{X}}_{N}{\hat{D}}_{N}=1,\;\;{\hat{D}}_{N}^{nm}=\frac{e^{-|n-m|ak}}{2k}.$$

Thus, ${\hat{X}}_{N}\left(k\right)$ can be found by inverting the matrix ${\hat{D}}_{N}^{nm}$,

(19) $${\hat{X}}_{N}\left(k\right)=\frac{2}{k}{\left(\begin{array}{ccccc} 1 & e^{-ak} & e^{-2ak} & ... & e^{-(N-1)ak}\\ e^{-ak} & 1 & e^{-ak} & ... & e^{-(N-2)ak}\\ e^{-2ak} & e^{-ak} & 1 & ... & e^{-(N-3)ak}\\ ... & ... & ... & ... & ...\\ e^{-(N-1)ak} & e^{-(N-2)ak} & e^{-(N-3)ak} & ... & 1 \end{array}\right)}^{-1}.$$

The matrix ${\hat{D}}_{N}^{nm}$ belongs to the class of symmetric Toeplitz matrices, the so-called Kac–Murdock–Szegö matrix [37]. One can use formulas available in the literature to invert such a matrix [38]. However, we find it more convenient to follow a different path.

We have found the matrix ${\hat{X}}_{2}\left(k\right)$ for the case of two planes N=2 in the previous subsection. To proceed, it makes sense to find the matrix ${\hat{X}}_{N}\left(k\right)$ for N=3 and then estimate its general form for the case of *N* planes. Later, we will confirm this prediction by using the general formula for the inverse of symmetric tridiagonal matrix. For N=3, we find

(20) $${\hat{X}}_{3}\left(k\right)=\frac{2}{k}{\left(\begin{array}{ccc} 1 & e^{-ak} & e^{-2ak}\\ e^{-ak} & 1 & e^{-ak}\\ e^{-2ak} & e^{-ak} & 1 \end{array}\right)}^{-1}=\frac{2}{k(1-e^{-2ak})}\left(\begin{array}{ccc} 1 & -e^{-ak} & 0\\ -e^{-ak} & 1+e^{-2ak} & -e^{-ak}\\ 0 & -e^{-ak} & 1 \end{array}\right).$$

Thus, the effective action for charged particles confined to three planes and interacting with gauge fields in the Feynman gauge takes the form

(21) $$\begin{array}{c} S_{\mathrm{eff}}^{F}=\int d^{3}x\left[\frac{1}{4}F_{\mu \nu }^{n}\;{\hat{X}}_{3}^{nm}\left(□\right)F_{\mu \nu }^{m}+\frac{1}{2}\left(\partial_{\mu }A_{\mu }^{n}\right){\hat{X}}_{3}^{nm}\left(□\right)\left(\partial_{\mu }A_{\mu }^{m}\right)+L_{\mathrm{int}}\right],\;\;\;n,m=1,2,3, \end{array}$$

where the operator form factor ${\hat{X}}_{3}$ is the 3×3 matrix

$$\begin{array}{c} {\hat{X}}_{3}\left(□\right)=\frac{2}{\sqrt{-□}\left(1-e^{-2a\sqrt{-□}}\right)}\left(\begin{array}{ccc} 1 & -e^{-a\sqrt{-□}} & 0\\ -e^{-a\sqrt{-□}} & 1+e^{-2a\sqrt{-□}} & -e^{-a\sqrt{-□}}\\ 0 & -e^{-a\sqrt{-□}} & 1 \end{array}\right) \end{array}$$

and $L_{\mathrm{int}}=A_{\mu }^{1}j_{\mu }^{1}+A_{\mu }^{2}j_{\mu }^{2}+A_{\mu }^{3}j_{\mu }^{3}$ describes the conventional interaction of vector gauge fields with charged particles.

Comparing expressions (16) and (20), we can predict that ${\hat{X}}_{N}\left(k\right)$ for the case of *N* planes has the form

(22) $${\hat{X}}_{N}\left(k\right)=\frac{2}{k(1-e^{-2ak})}\left(\begin{array}{ccccc} 1 & -e^{-ak} & 0 & ... & 0\\ -e^{-ak} & 1+e^{-2ak} & -e^{-ak} & ... & 0\\ 0 & -e^{-ak} & 1+e^{-2ak} & ... & 0\\ ... & ... & ... & ... & -e^{-ak}\\ 0 & 0 & ... & -e^{-ak} & 1 \end{array}\right).$$

To prove this prediction, note that ${\hat{X}}_{N}$ is a symmetric tridiagonal matrix. The general formula for the inverse of a symmetric tridiagonal matrix is provided by Theorem 2.3 in [39]. A symmetric tridiagonal matrix has the following general form:

$$T=\left(\begin{array}{ccccc} a_{1} & -b_{2} & & & \\ -b_{2} & a_{2} & -b_{3} & & \\ & ... & ... & ... & \\ & & -b_{n-1} & a_{n-1} & -b_{n}\\ & & & -b_{n} & a_{n} \end{array}\right),$$

where all elements of *T* outside the three diagonals are zero. In terms of quantities

$$\delta_{1}=a_{1},\;\delta_{i}=a_{i}-\frac{b_{i}^{2}}{\delta_{i-1}},\;i=2,...,n$$

and

$$d_{n}=a_{n},\;d_{i}=a_{i}-\frac{b_{i+1}^{2}}{d_{i+1}},\;i=n-1,...,1,$$

the diagonal and off-diagonal elements of the matrix $T^{-1}$ are given by

$$T_{ii}^{-1}=\frac{d_{i+1}...d_{n}}{\delta_{i}...\delta_{n}},$$

$$T_{ij}^{-1}=b_{i+1}...b_{j}\frac{d_{j+1}...d_{n}}{\delta_{i}...\delta_{n}},\;j>i.$$

By using the above formula, one can easily check that ${\left({\hat{X}}_{N}\right)}^{-1}$, where ${\hat{X}}_{N}$ is given by Equation (22), indeed equals ${\hat{D}}_{N}$.

## 3. Screened Interaction

Let us determine how the screening effects modify the electron–electron interactions in a heterostructure with *N* equivalent planes. The screened interaction is defined by the well-known equation

$${\left({\hat{D}}_{N,\;scr}\right)}^{-1}=k^{2}{\hat{X}}_{N}+\hat{\Pi }\left(k\right),$$

i.e.,

(23) $${\hat{D}}_{N,\;scr}={\left(k^{2}{\hat{X}}_{N}+\hat{\Pi }\left(k\right)\right)}^{-1},$$

where $\hat{\Pi }\left(k\right)$ is the polarization function due to charged fermions. In order to use the derivation of ${\hat{D}}_{N}$ in the previous section by applying the general formula for the inverse of symmetric tridiagonal matrix, it is convenient to rewrite (23) as follows:

(24) $$k{\hat{D}}_{N,\;scr}={\left(k{\hat{X}}_{N}+\frac{\hat{\Pi }}{k}\right)}^{-1}.$$

In the simplest case of two planes, N=2, assuming that the polarization function is a diagonal matrix in plane indices with different planes’ polarizations, $\hat{\Pi }=diag\left(\Pi_{1},\Pi_{2}\right)$, we find

(25) $$\begin{array}{cc} & D_{scr}^{11}\left(k\right)=\frac{1}{2k}\frac{1+\frac{\left(1-e^{-2ak}\right)\Pi_{2}}{2k}}{\left(1+\frac{\Pi_{1}}{2k}\right)\left(1+\frac{\Pi_{2}}{2k}\right)-\frac{e^{-2ak}\Pi_{1}\Pi_{2}}{4k^{2}}},\;D_{scr}^{22}\left(k\right)=\frac{1}{2k}\frac{1+\frac{\left(1-e^{-2ak}\right)\Pi_{1}}{2k}}{\left(1+\frac{\Pi_{1}}{2k}\right)\left(1+\frac{\Pi_{2}}{2k}\right)-\frac{e^{-2ak}\Pi_{1}\Pi_{2}}{4k^{2}}},\\ \; & D_{scr}^{12}\left(k\right)=D_{scr}^{21}\left(k\right)=\frac{1}{2k}\frac{e^{-ak}}{\left(1+\frac{\Pi_{1}}{2k}\right)\left(1+\frac{\Pi_{2}}{2k}\right)-\frac{e^{-2ak}\Pi_{1}\Pi_{2}}{4k^{2}}}, \end{array}$$

which agrees with Ref. [40] (Equation (S11) in the Supplemental Materials). In the next section, we will study the gap generation in a heterostructure composed of two equivalent planes. Therefore, we will need formulas for the screened interaction with the same polarization in the two planes, $\Pi_{1}=\Pi_{2}=\Pi$. In this case, the photon propagator takes the simpler form:

(26) $$\begin{array}{c} D_{scr}\left(k\right)=\frac{1}{2k}\frac{1}{{\left(1+\frac{\Pi }{2k}\right)}^{2}-\frac{e^{-2ak}\Pi^{2}}{4k^{2}}}\left(\begin{array}{cc} 1+\frac{(1-e^{-2ak})\Pi }{2k}\; & e^{-ak}\\ e^{-ak} & 1+\frac{(1-e^{-2ak})\Pi }{2k} \end{array}\right). \end{array}$$

Thus, we obtained the explicit expressions for the effective screened interaction in the case of two planes. In Appendix A, we present the corresponding expressions for the effective screened interaction with three non-equivalent and equivalent polarization functions in Equations (A1) and (A3), respectively. By using the general formula for the inverse of a symmetric tridiagonal matrix, one can find the effective screened interaction for any *N*.

It is also of interest to consider the more general case of non-diagonal polarization, for example,

(27) $$\begin{array}{c} \hat{\Pi }=\left(\begin{array}{cc} \Pi_{s} & \Pi_{d}\\ \Pi_{d} & \Pi_{s} \end{array}\right), \end{array}$$

with equal polarization function $\Pi_{s}$ in the same layer and the polarization function $\Pi_{d}$ for different layers, where charged fermions in different planes influence each other [36]. Using Equation (24), we find

(28) $$\begin{array}{cc} \; & D_{scr}^{11}\left(k\right)=D_{scr}^{22}\left(k\right)=\frac{1}{2k}\frac{1+\left(1-e^{-2ak}\right)\frac{\Pi_{s}}{2k}}{\left[1+\left(1+e^{-ak}\right)\frac{\Pi_{s}+\Pi_{d}}{2k}\right]\left[1+\left(1-e^{-ak}\right)\frac{\Pi_{s}-\Pi_{d}}{2k}\right]}, \end{array}$$

(29) $$\begin{array}{cc} \; & D_{scr}^{12}\left(k\right)=D_{scr}^{21}\left(k\right)=\frac{1}{2k}\frac{e^{-ak}-\left(1-e^{-2ak}\right)\frac{\Pi_{d}}{2k}}{\left[1+\left(1+e^{-ak}\right)\frac{\Pi_{s}+\Pi_{d}}{2k}\right]\left[1+\left(1-e^{-ak}\right)\frac{\Pi_{s}-\Pi_{d}}{2k}\right]}. \end{array}$$

These equations agree with Equations (9) and (10) in [41] in the case of two layers. One can also check that Equation (29) is in agreement with Equation (5) in [36] (except of a minus sign due to a different definition of the polarization functions). Of course, Equations (28) and (29) reduce to Equation (26) for $\Pi_{d}=0$.

## 4. Gap Equation for Double-Layer Graphene

As an example of the application of the obtained formulas for reduced QED, extended to the case of several planes, let us consider the gap generation in a heterostructure with two equivalent planes. Its charge carriers like in graphene, or in topological insulator surface layers, are described by the relativistic-like massless Dirac equation. The corresponding free inverse propagator for these charged particles with the same chemical potential μ in two planes is given by (we set the Planck constant ħ=1)

(30) $${\hat{S}}^{-1}\left(\omega ,p\right)=-i\delta_{nm}\left(\left(i\omega -\mu \right)\gamma^{0}+v_{F}p\gamma \right)=\delta_{nm}S^{-1},$$

where $v_{F}$ is the Fermi velocity; *m* and *n* are the indices of planes, which take values 1 and 2; and $\gamma^{\mu }=\left(\gamma^{0},\gamma \right)$ are the 4×4 Dirac matrices furnishing, like in graphene, a reducible representation of the Dirac algebra in (2+1) dimensions. These fermions interact with the electromagnetic field via the usual $A_{\mu }j^{\mu }$ term, where $j^{\mu }=\left(\rho ,j\right)$ with $\rho =e\bar{\psi }\gamma^{0}\psi$ and $j=ev_{F}\bar{\psi }\gamma \psi$. Here, ψ is the four-component spinor field and $\bar{\psi }=\psi^{†}\gamma^{0}$. Since, typically, the Fermi velocity $v_{F}$ is much less than the speed of light, we take into account in our analysis of the gap generation only the Coulomb interaction term $\rho A_{0}$. Then, the Schwinger–Dyson equation for the fermion propagator $\hat{G}$ at temperature *T* has the form

(31) $${\hat{G}}^{-1}\left(\omega_{m},p\right)={\hat{S}}^{-1}\left(\omega_{m},p\right)-e^{2}T\sum_{n=-\infty }^{+\infty }\int \frac{d^{2}k}{{\left(2\pi \right)}^{2}}\left(\gamma^{0}⊗I_{2}\right)\hat{G}\left(\omega_{n},k\right)\left(\gamma^{0}⊗I_{2}\right){\hat{D}}_{2,\;scr}\left(p-k\right),$$

where $\omega_{m}=\left(2m+1\right)\pi T$ are the fermion Matsubara frequencies with integer *m*, $I_{2}$ is the 2×2 unit matrix in plane indices, and the elements of the screened static interaction ${\hat{D}}_{2,\;scr}\left(k\right)$ are given in Equation (26). We use the bare vertex approximation; for effects (−e<0 is the electron charge) of vertex corrections, see Ref. [42] and references therein.

To uncover the possible types of the gap, it is useful to represent the full inverse fermion propagator in the block form

(32) $${\hat{G}}^{-1}=\left(\begin{array}{cc} A & B\\ C & D \end{array}\right),$$

where *A*, *B*, *C*, and *D* are 4×4 matrices. One can distinguish three types of gaps: (i) diagonal gap (like in graphene) Δ with $A=D=S^{-1}+\Delta$ and B=C=0; (ii) off-diagonal gap *m* with $A=D=S^{-1}$ and B=C=m; and (iii) the general case with $A=S^{-1}+\Delta$, $D=S^{-1}-\Delta$, and B=C=m.

### 4.1. Diagonal Gap

This is the simplest case for analysis. Neglecting the wave function renormalization and using Equation (31), we obtain the following gap equation (compare this equation with Equation (B8) in [8]):

(33) $$\Delta \left(p\right)=\frac{e^{2}}{4}\int \frac{d^{2}k}{{\left(2\pi \right)}^{2}}\frac{\Delta (k)}{\varepsilon_{k}}\frac{\sinh\frac{\varepsilon_{k}}{T}}{\sinh\frac{\varepsilon_{k}}{T}+\cosh\frac{\mu }{T}}\times \frac{1}{\left|p-k\right|}\frac{1+\frac{(1-e^{-2a|p-k|})\Pi }{2|p-k|}}{{\left(1+\frac{\Pi }{2|p-k|}\right)}^{2}-\frac{e^{-2a|p-k|}\Pi^{2}}{{4|p-k|}^{2}}},$$

where $\varepsilon_{k}=\sqrt{v_{F}^{2}k^{2}+\Delta^{2}}$. For a→∞, this screened interaction tends to that in graphene. Denoting $x=\frac{e^{-2a|p-k|}\Pi }{2|p-k|}$ and expanding the interaction in *x*, we find that the first correction in *x*

$$\frac{1}{|p-k|+\frac{1}{2}\Pi \left(0,p-k\right)}\left(1-\frac{x}{1+\frac{\Pi }{2|p-k|}}\right)$$

is negative, i.e., the effective strength of interaction decreases compared to that in graphene.

For different planes with different polarization functions $\Pi_{1}$ and $\Pi_{2}$, one can show that the interaction strength increases if $\Pi_{1}$ or $\Pi_{2}$ decreases.

### 4.2. Off-Diagonal Gap

For the off-diagonal gap, by using the formula for blockwise inversion, we find that Equation (32) results in:

$$\hat{G}=\left(\begin{array}{cc} A^{-1}+A^{-1}B{\left(D-CA^{-1}B\right)}^{-1}CA^{-1} & -A^{-1}B{\left(D-CA^{-1}B\right)}^{-1}\\ -{\left(D-CA^{-1}B\right)}^{-1}CA^{-1} & {\left(D-CA^{-1}B\right)}^{-1} \end{array}\right).$$

Since matrices *C* and *B* commute with *A* and *D* in our case and ignoring again the wave function renormalization, we find that Equation (31) implies the following gap equation:

(34) $$m\left(p\right)=\frac{e^{2}}{4}\int \frac{d^{2}k}{{\left(2\pi \right)}^{2}}\frac{m(k)}{\epsilon_{k}}\frac{\sinh\frac{\epsilon_{k}}{T}}{\sinh\frac{\epsilon_{k}}{T}+\cosh\frac{\mu }{T}}\times \frac{1}{\left|p-k\right|}\frac{e^{-a|p-k|}}{{\left(1+\frac{\Pi }{\left|p-k\right|}\right)}^{2}-\frac{e^{-2a|p-k|}\Pi^{2}}{{\left|p-k\right|}^{2}}},$$

where $\epsilon_{k}=\sqrt{v_{F}^{2}k^{2}+m^{2}}$. Let us compare Equations (33) and (34). Since

$$e^{-a|p-k|}<1+\frac{(1-e^{-2a|p-k|})\Pi }{2|p-k|},$$

this inequality means that the kernel of the gap equation for the off-diagonal gap is smaller than the kernel for the diagonal gap. Hence, the critical coupling constant for the diagonal gap generation will be smaller than that for the off-diagonal gap. Thus, we conclude that the generation of the off-diagonal gap is less favorable than the diagonal one.

### 4.3. General Case

The gap equations in this case form a system of two connected equations for Δ and *m*

(35) $$\Delta \left(p\right)=\frac{e^{2}}{4}\int \frac{d^{2}k}{{\left(2\pi \right)}^{2}}\frac{\Delta (k)}{E_{k}}\frac{\sinh\frac{E_{k}}{T}}{\sinh\frac{E_{k}}{T}+\cosh\frac{\mu }{T}}\times \frac{1}{\left|p-k\right|}\frac{1+\frac{(1-e^{-2a|p-k|})\Pi }{2|p-k|}}{{\left(1+\frac{\Pi }{2|p-k|}\right)}^{2}-\frac{e^{-2a|p-k|}\Pi^{2}}{{4|p-k|}^{2}}},$$

(36) $$m\left(p\right)=\frac{e^{2}}{4}\int \frac{d^{2}k}{{\left(2\pi \right)}^{2}}\frac{m(k)}{E_{k}}\frac{\sinh\frac{E_{k}}{T}}{\sinh\frac{E_{k}}{T}+\cosh\frac{\mu }{T}}\times \frac{1}{\left|p-k\right|}\frac{e^{-a|p-k|}}{{\left(1+\frac{\Pi }{2|p-k|}\right)}^{2}-\frac{e^{-2a|p-k|}\Pi^{2}}{{4|p-k|}^{2}}},$$

where $E_{k}=\sqrt{v_{F}^{2}k^{2}+\Delta^{2}+m^{2}}$. For μ=0 and T→0, we have

$$\frac{1}{E_{k}}\frac{\sinh\frac{E_{k}}{T}}{\sinh\frac{E_{k}}{T}+\cosh\frac{\mu }{T}}\;\to \;\frac{1}{E_{k}}.$$

The energy dispersion $E_{k}$ is present in denominators of the integrands of the gap equations and increases with Δ and *m*. Since the rest of the integrands coincides with that of the gap equations for Δ and *m* considered in Section 4.1 and Section 4.2, respectively, we conclude that the generation of two non-zero gaps is not favorable compared to the case of the gap generation of one type.

## 5. Gap Generation and Critical Coupling Constant

We argued in the previous section that the interaction is stronger for the diagonal gap Δ compared to the case of the off-diagonal gap *m*. Therefore, we will solve in this section only the gap equation for the diagonal gap Δ and determine the dependence of the critical coupling constant for the onset of a gap on the interplane distance *a* at zero chemical potential μ=0 and temperature T=0. As in [8], we consider the random phase approximation where the polarization function is given by the one-loop expression with massless fermions

(37) $$\Pi \left(0,k\right)=\frac{e^{2}N_{f}}{8v_{F}}\left|k\right|,$$

where $N_{f}$ is the number of charged fermion species. The use of the polarization with massless fermions is justified since the region $\left|k\right|\gg \Delta /v_{F}$ dominates in the integral equation [8]. Moreover, since we are interested in finding the critical coupling constant, near which Δ is close to zero, such an approximation is well justified.

Taking into account the polarization function (37), the gap equation for the diagonal gap Δ takes the from

(38) $$\Delta \left(p\right)=\frac{e^{2}}{4}\int \frac{d^{2}k}{{\left(2\pi \right)}^{2}}\frac{\Delta (k)}{\varepsilon_{k}}K(|p-k|),\;\;K(|p-k|)=\frac{1}{\left|p-k\right|}\frac{1+(1-e^{-2a|p-k|})r}{{\left(1+r\right)}^{2}-e^{-2a|p-k|}r^{2}},$$

where $r=\frac{e^{2}N_{f}}{16v_{F}}$. Using the standard approximation f(|p−k|)→f(p)θ(p−k)+f(k)θ(k−p) for the kernel K(|p−k|) and integrating over angle, we obtain

(39) $$\Delta \left(p\right)=\frac{e^{2}}{8\pi v_{F}}\int_{0}^{\Lambda }\frac{dkk\Delta (k)}{\sqrt{k^{2}+{\left(\Delta_{k}/v_{F}\right)}^{2}}}K\left(p,k\right)\;,$$

where the new kernel K(p,k) is given by the expression

(40) $$K\left(p,k\right)=\theta \left(p-k\right)f\left(p\right)+\theta \left(k-p\right)f\left(k\right),\;f\left(p\right)=\frac{1}{p}\frac{1+(1-e^{-2ap})r}{{\left(1+r\right)}^{2}-e^{-2ap}r^{2}}$$

and we introduce an ultraviolet cut-off Λ.

Clearly, the gap equatrion has the trivial solution Δ(p)=0, but we are interested in the nontrivial one. The term ${\left(\Delta_{k}/v_{F}\right)}^{2}$ in the denominator provides an IR cut-off. In the bifurcation approximation, we drop this term and introduce an explicit IR cut-off in the integral for which we take the value of the gap function at zero momentum $\Delta_{0}\equiv \Delta_{p=0}$. We obtain

(41) $$\Delta_{p}=\frac{e^{2}}{8\pi v_{F}}\left(f\left(p\right)\int_{\Delta_{0}/v_{F}}^{p}dk\;\Delta_{k}+\int_{p}^{\Lambda }dk\;\Delta_{k}f\left(k\right)\right).$$

The latter integral equation is equivalent to the differential equation

(42) $$\Delta_{p}^{\prime \prime }-\frac{\Delta_{p}^{\prime }f^{\prime \prime }}{f^{\prime }}-\frac{e^{2}}{8\pi v_{F}}\Delta_{p}f^{\prime }=0,$$

with the boundary conditions

(43) $$\begin{array}{c} \frac{\Delta_{p}^{\prime }}{f^{\prime }}{|}_{p=\frac{\Delta_{0}}{v_{F}}}=0,\\ {\left(\frac{\Delta_{p}}{f}\right)}^{\prime }{|}_{p=\Lambda }=0. \end{array}$$

Since the function *f* in Equation (40) equals $f=\frac{1}{p(1+2r)}$ for p≪1/2a and $f=\frac{1}{p(1+r)}$ for p≫1/2a, we can solve the gap equation in the corresponding asymptotic regions and then match solutions at the point $p=\frac{1}{2a}$.

The differential Equation (42) for p≪1/2a is similar to that in graphene

(44) $$p^{2}\Delta_{p}^{\prime \prime }+2p\Delta_{p}^{\prime }+\lambda_{1}\Delta_{p}=0,$$

where

(45) $$\lambda_{1}=\frac{(1+r)\lambda }{1+2r}.$$

In graphene, $\lambda_{1}$ is replaced by λ with

(46) $$\lambda =\frac{e^{2}}{8\pi v_{F}\left(1+e^{2}N_{f}/\left(16v_{F}\right)\right)}.$$

The IR boundary condition (43) for $f=\frac{1}{p(1+2r)}$ takes the form

$$\Delta_{p}^{\prime }{|}_{p=\frac{\Delta_{0}}{v_{F}}}=0.$$

The solution $\Delta_{1}$ at small momenta, which satisfies the IR boundary condition and equals $\Delta_{1}\left(\Delta_{0}/v_{F}\right)=\Delta_{0}$, is given by

(47) $$\Delta_{1}\left(p\right)=\frac{\Delta_{0}^{3/2}}{\sin\left(\delta_{1}\right)\sqrt{pv_{F}}}\sin\left(\frac{\sqrt{4\lambda_{1}-1}}{2}\ln\frac{pv_{F}}{\Delta_{0}}+\delta_{1}\right),$$

where $\delta_{1}=\mathrm{artan}\sqrt{4\lambda_{1}-1}$ with $\lambda_{1}=\frac{e^{2}}{8\pi v_{F}\left(1+2r\right)}$. It is not difficult to find solution $\Delta_{2}$ at large momenta, which equals

(48) $$\Delta_{2}\left(p\right)=\frac{\Delta_{0}^{3/2}C_{2}}{\sin\left(\delta_{1}\right)\sqrt{pv_{F}}}\sin\left(\frac{\sqrt{4\lambda -1}}{2}\ln\frac{pv_{F}}{\Delta_{0}}+\delta_{2}\right),$$

where $C_{2}$ and $\delta_{2}$ are arbitrary constants.

The matching conditions at $p=\frac{1}{2a}$,

$$\Delta_{1}\left(1/2a\right)=\Delta_{2}\left(1/2a\right),\;\;\Delta_{1}^{\prime }\left(1/2a\right)=\Delta_{2}^{\prime }\left(1/2a\right),$$

determine the constant

$$C_{2}=\frac{\sin\left(\frac{\sqrt{4\lambda_{1}-1}}{2}\ln\frac{v_{F}}{2a\Delta_{0}}+\delta_{1}\right)}{\sin\left(\frac{\sqrt{4\lambda -1}}{2}\ln\frac{v_{F}}{2a\Delta_{0}}+\delta_{2}\right)}$$

and result in the equation for $\delta_{2}$:

(49) $$\tan\left(\frac{\sqrt{4\lambda -1}}{2}\ln\frac{v_{F}}{2a\Delta_{0}}+\delta_{2}\right)=\frac{\sqrt{4\lambda -1}}{\sqrt{4\lambda_{1}-1}}\tan\left(\frac{\sqrt{4\lambda_{1}-1}}{2}\ln\frac{v_{F}}{2a\Delta_{0}}+\delta_{1}\right).$$

The UV boundary condition (43) equals

$$\frac{\Delta_{2}^{\prime }\left(\Lambda \right)}{\Delta_{2}\left(\Lambda \right)}=-\frac{1}{\Lambda }$$

and results in the equation

(50) $$\frac{\sqrt{4\lambda -1}}{2}\ln\frac{\Lambda v_{F}}{\Delta_{0}}+\delta_{2}+\delta =\pi ,$$

where $\delta =\arctan\sqrt{4\lambda -1}$. Finding the phase $\delta_{2}$ from Equation (50) and plugging it into Equation (49), we arrive at the equation for $\Delta_{0}$:

(51) $$\begin{array}{c} \tan\left(\frac{\sqrt{4\lambda -1}}{2}\ln\left(2a\Lambda \right)+\delta \right)=-\frac{\sqrt{4\lambda -1}}{\sqrt{4\lambda_{1}-1}}\tan\left(\frac{\sqrt{4\lambda_{1}-1}}{2}\ln\frac{v_{F}}{2a\Delta_{0}}+\delta_{1}\right). \end{array}$$

According to the bifurcation theory, the limit $\Delta_{0}\to 0$ determines the critical value of the coupling constant at which the nontrivial solution for the gap branches off from the trivial solution. Obviously, the limit $\Delta_{0}\to 0$ in Equation (51) exists only for values $\lambda_{1}<1/4$ and, for $a\Delta_{0}\ll 1$, the equation takes the form

(52) $$\tan\left(\frac{\sqrt{4\lambda -1}}{2}\ln\left(2a\Lambda \right)+\delta \right)=-\frac{\sqrt{4\lambda -1}}{\sqrt{1-4\lambda_{1}}}\left(1-2e^{-2d_{1}}{\left(\frac{2a\Delta_{0}}{v_{F}}\right)}^{\sqrt{1-4\lambda_{1}}}\right),$$

where $d_{1}=\mathrm{artanh}\sqrt{1-4\lambda_{1}}$. Or, equivalently,

(53) $$\Delta_{0}=\frac{v_{F}}{2a}{\left[\frac{e^{2d_{1}}}{2}\left(1+\frac{\sqrt{1-4\lambda_{1}}}{\sqrt{4\lambda -1}}\tan\left(\frac{\sqrt{4\lambda -1}}{2}\ln\left(2a\Lambda \right)+\delta \left(\lambda \right)\right)\right)\right]}^{\frac{1}{\sqrt{1-4\lambda_{1}}}}.$$

For $\Delta_{0}=0$, we find the equation which determines the critical coupling constant $\lambda_{\mathrm{cr}}$,

(54) $$\frac{\sqrt{4\lambda_{\mathrm{cr}}-1}}{2}\ln\left(2a\Lambda \right)+\arctan\left(\frac{\sqrt{4\lambda_{\mathrm{cr}}-1}}{\sqrt{1-4\lambda_{1}}}\right)+\delta \left(\lambda_{\mathrm{cr}}\right)=\pi .$$

It is useful to recall the gap equation in graphene

(55) $$\frac{\sqrt{4\lambda -1}}{2}\ln\frac{\Lambda v_{F}}{\Delta_{0}}+2\delta =\pi$$

which has a similar form and provides the critical coupling constant $\lambda_{gr,\;cr}=1/4$. The approximate solution to (54) for aΛ≫1 is given by

(56) $$\lambda_{\mathrm{cr}}\approx \frac{1}{4}\left(1+{\left(\frac{2\pi }{\ln(2a\Lambda )}\right)}^{2}\right),$$

which is larger than the critical coupling constant $\lambda_{gr,\;cr}=1/4$ in graphene. Using $\lambda =\lambda_{\mathrm{cr}}+\delta \lambda$, $\delta \lambda =\lambda -\lambda_{\mathrm{cr}}$, we obtain that in view of Equation (53), the gap scales near the critical coupling constant as follows:

(57) $$\Delta_{0}\left(\lambda \right)=\frac{v_{F}}{2a}{\left[\frac{e^{2d_{1}}}{4\lambda_{\mathrm{cr}}-1}\left(1+\frac{2(\lambda_{\mathrm{cr}}-\lambda_{1})}{\sqrt{1-4\lambda_{1}}}\left(\ln\left(2a\Lambda \right)+\frac{1}{2\lambda_{\mathrm{cr}}}\right)\right)\left(\lambda -\lambda_{\mathrm{cr}}\right)\right]}^{\frac{1}{\sqrt{1-4\lambda_{1}}}}.$$

In the case of the critical coupling (56), this expression simplifies when ln(2aΛ)≫1 and takes the form

(58) $$\Delta_{0}=\frac{v_{F}}{2a}{\left[\frac{e^{2d_{1}}\left(\lambda_{\mathrm{cr}}-\lambda_{1}\right)}{2\pi^{2}\sqrt{1-4\lambda_{1}}}\ln^{3}\left(2a\Lambda \right)\;\left(\lambda -\lambda_{\mathrm{cr}}\right)\right]}^{\frac{1}{\sqrt{1-4\lambda_{1}}}}.$$

**Figure 1:**
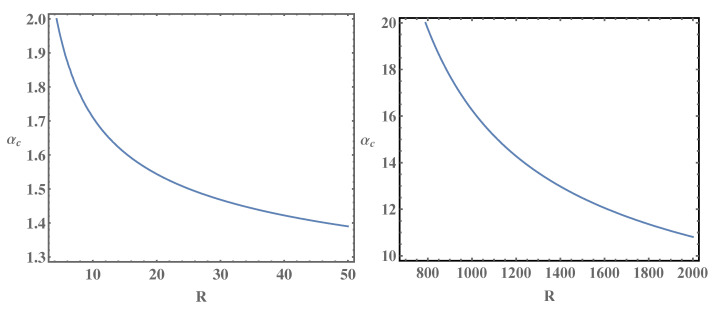
The critical Coulomb coupling $\alpha_{c}$ for $N_{f}=1$ (**left panel**) and $N_{f}=2$ (**right panel**) as a function of distance $R=a/a_{0}$ (in terms of the lattice constant $a_{0}$) between planes.

To present the physical value of the wave vector cut-off Λ, we relate it to the graphene lattice constant $a_{0}$ by means of the formula $\Lambda ={\left(4\pi /\sqrt{3}\right)}^{1/2}\;/a_{0}$ [43]; hence, $\ln\left(2a\Lambda \right)=\ln\left(5.4a/a_{0}\right)$. Introducing $R=a/a_{0}$, we find that Equation (54) determines the sought dependence of the critical Coulomb coupling

(59) $$\alpha_{c}=\frac{e^{2}}{4\pi v_{F}}=\frac{2\lambda_{\mathrm{cr}}}{1-\frac{\lambda_{\mathrm{cr}}\pi N_{f}}{2}}$$

on the distance between planes, which is shown in Figure 1 for $N_{f}=1$ (left panel) and $N_{f}=2$ (right panel). For $N_{f}=2$, the values of the critical coupling $\alpha_{c}$ are much larger (notice the difference in scales in left and right panels). We remind that for the single-layer graphene in the same approximation, we have $\alpha_{c}=0.82$ ($N_{f}=1$) and $\alpha_{c}=2.33$ ($N_{f}=2$) [8]. More refined approximations for the kernel of the integral gap equation, and taking into account the frequency-dependent polarization, usually significantly reduce the value $\alpha_{c}$ [18]. The second sheet increases the screening of the electron–electron interaction since due to its presence, the polarization function acquires an additional contribution. The larger screening means that the kernel of the gap equation is reduced. Hence, a larger critical coupling is needed for the gap generation. Thus, the presence of the second sheet leads to an increase in the value $\alpha_{c}$, which in this case depends on the distance between sheets.

## 6. Summary

The effective (2+1)-dimensional theory for charged particles confined to *N* planes was formulated. Such a dimensionally reduced theory contains *N* vector fields with Maxwell’s action modified by non-local form factors whose explicit form is determined. This theory extends the formalism of reduced QED to the case of multilayer structures. It could also be useful and efficient for the study of heterostructures composed via van der Waals’ assembly of 2D crystals. Taking into account the polarization function, the explicit formulae for screened interaction in the reduced theory were presented in the case of two and three layers. A polarization matrix, which is nondiagonal in layer indices, allows us to account for the case of charged planes.

By using the extended formalism of the reduced QED theory for a nanostructure composed of two equivalent layers and charged fermions described by the massless Dirac equation, we studied the dynamical gap generation considering two types of gaps. While one of them is similar to that in graphene, the other describes interlayer coherence. Using the Schwinger–Dyson equations, and taking into account the polarization function in the static approximation, we derived the corresponding gap equations. Upon solving them in the random phase approximation, we found that the generation of the gap similar to that in graphene is favorable. However, the additional screening due to the presence of the second layer increases the value of the critical coupling constant compared to that in graphene. Since dynamical screening diminishes the polarization function, the critical coupling constant for the dynamical gap generation should decrease in the case of the dynamical polarization function as is known from previous studies [18,20].

As is known, experimental measurements [44] indicate the absence of a gap in the quasiparticle spectrum of suspended graphene, which can be explained by the additional screening of the Coulomb interaction due to the σ bands and the renormalization of the fermion velocity (see discussion in Ref. [21] and references therein). Additional conducting planes, which could be present in experimental setups not far from the graphene sheet, might be another reason for the absence of the gap generation in suspended graphene, like in the case considered in the present paper. An interesting possibility for the application of the developed formalism of reduced QED with few planes is the study of the pairing of electrons and holes from different oppositely charged layers [36].

## Data Availability

Not applicable.

## Appendix A. Effective Screened Interaction for Three Planes

The effective screened interaction for three non-equivalent planes is given by:

(A1) $$\begin{array}{cc} \; & {\hat{D}}_{3,scr}\left(k\right)=\frac{x}{2kD_{n}}\times \\ \; & \left(\begin{array}{ccc} \left(1+p_{3}x\right)\left(1+p_{2}x\right)+p_{3}xe^{-2ak} & \left(1+p_{3}x\right)e^{-ak} & e^{-2ak}\\ \left(1+p_{3}x\right)e^{-ak} & \left(1+p_{1}x\right)\left(1+p_{3}x\right) & \left(1+p_{1}x\right)e^{-ak}\\ e^{-2ak} & \left(1+p_{1}x\right)e^{-ak} & \left(1+p_{1}x\right)\left(1+p_{2}x\right)+p_{1}xe^{-2ak} \end{array}\;\right), \end{array}$$

where

(A2) $$\begin{array}{cc} D_{n}= & \left(1+p_{3}x\right)\left[\left(1+p_{1}x\right)\left(1+p_{2}x+e^{-2ak}\right)-e^{-2ak}\right]-e^{-2ak}\left(1+p_{1}x\right),\\ \; & p_{i}=\frac{\Pi_{i}\left(k\right)}{2k},\;i=\bar{1,3},\;x=1-e^{-2ak}. \end{array}$$

For three equivalent planes with $\Pi_{1}=\Pi_{2}=\Pi_{3}=\Pi$, we find a simpler expression for the effective screened interaction

(A3) $${\hat{D}}_{3,\;scr}\left(k\right)=\frac{x}{2kD_{e}}\left(\begin{array}{cccc} {\left(1+px\right)}^{2}+pxe^{-2ak} & \left(1+px\right)e^{-ak} & e^{-2ak}\\ \left(1+px\right)e^{-ak} & {\left(1+px\right)}^{2} & \left(1+px\right)e^{-ak} & \\ e^{-2ak} & \left(1+px\right)e^{-ak} & {\left(1+px\right)}^{2}+pxe^{-2ak} \end{array}\right),$$

where

$$D_{e}=\left(1+px\right)\left[{\left(1+px\right)}^{2}-\left(1-px\right)e^{-2ak}\right],\;p=\frac{\Pi (k)}{2k},\;x=1-e^{-2ak}.$$

## References

[1] Gorbar E.V., Gusynin V.P., Miransky V.A. (2001). Dynamical chiral symmetry breaking on a brane in reduced QED. Phys. Rev. D.

[2] Marino E.C. (1993). Quantum electrodynamics of particles on a plane and the Chern-Simons theory. Nucl. Phys. B.

[3] Kovner A., Rosenstein B. (1990). Kosterlitz-Thouless mechanism of two-dimensional superconductivity. Phys. Rev. B.

[4] Dorey N., Mavromatos N.E. (1992). QED_3_ and two-dimensional superconductivity without parity violation. Nucl. Phys. B.

[5] Kotikov A.V., Teber S. (2014). Two-loop fermion self-energy in reduced quantum electrodynamics and application to the ultrarelativistic limit of graphene. Phys. Rev. D.

[6] Rubakov V.A., Shaposhnikov M.E. (1983). Do we live inside a domain wall?. Phys. Lett. B.

[7] Randall L., Sundrum R. (1999). Large Mass Hierarchy from a Small Extra Dimension. Phys. Rev. Lett..

[8] Gorbar E.V., Miransky V.A., Gusynin V.P., Shovkovy I.A. (2002). Magnetic field driven metal-insulator phase transition in planar systems. Phys. Rev. B.

[9] Hasan M.Z., Moore J.E. (2011). Three-Dimensional Topological Insulators. Annu. Rev. Cond. Mat. Phys..

[10] Polini M., Guinea F., Lewenstein M., Manoharan H.C., Pellegrini V. (2013). Artificial honeycomb lattices for electrons, atoms and photons. Nat. Nanotechnol..

[11] Marino E.C., Nascimento L.O., Alves V.S., Smith C.M. (2014). Unitarity of theories containing fractional powers of the d’Alembertian operator. Phys. Rev. D.

[12] Marino E.C., Nascimento L.O., Alves V.S., Menezes N., Smith C.M. (2018). Quantum-electrodynamical approach to the exciton spectrum in transition-metal dichalcogenides. 2D Mater..

[13] Fernández L., Alves V.S., Nascimento L.O., Pena F., Gomes M., Marino E.C. (2020). Renormalization of the band gap in 2D materials through the competition between electromagnetic and four-fermion interactions in large *N* expansion. Phys. Rev. D.

[14] Menezes N., Palumbo G., Smith C.M. (2017). Conformal QED in two-dimensional topological insulators. Sci. Rep..

[15] Dudal D., Mizher A.J., Pais P. (2019). Exact quantum scale invariance of three-dimensional reduced QED theories. Phys. Rev. D.

[16] Khveshchenko D.V. (2001). Ghost Excitonic Insulator Transition in Layered Graphite. Phys. Rev. Lett..

[17] Khveshchenko D.V., Leal H. (2004). Excitonic instability in layered degenerate semimetals. Nucl. Phys. B.

[18] Gamayun O.V., Gorbar E.V., Gusynin V.P. (2010). Gap generation and semimetal-insulator phase transition in graphene. Phys. Rev. B.

[19] Liu G.-Z., Wang J.-R. (2011). Competition between excitonic gap generation and disorder scattering in graphene. New J. Phys..

[20] Wang J.-R., Liu G.-Z. (2011). Dynamic gap generation in graphene under the long-range Coulomb interaction. J. Phys. Cond. Matter.

[21] Popovici C., Fischer C.S., von Smekal L. (2013). Fermi velocity renormalization and dynamical gap generation in graphene. Phys. Rev. B.

[22] Drut J.E., Lähde T.A. (2009). Lattice field theory simulations of graphene. Phys. Rev. B.

[23] Buividovich P., Smith D., Ulybyshev M., von Smekal L. (2019). Numerical evidence of conformal phase transition in graphene with long-range interactions. Phys. Rev. B.

[24] Kotikov A., Teber S. (2016). Critical behavior of reduced QED_4,3_ and dynamical fermion gap generation in graphene. Phys. Rev. D.

[25] Teber S., Kotikov A.V. (2018). Field theoretic renormalization study of reduced quantum electrodynamics and applications to the ultrarelativistic limit of Dirac liquids. Phys. Rev. D.

[26] Alves V.S., Reginaldo O.C., Marino E.C., Nascimento L.O. (2017). Dynamical mass generation in pseudoquantum electrodynamics with four-fermion interactions. Phys. Rev. D.

[27] Teber S. (2018). Field theoretic study of electron-electron interaction effects in Dirac liquids. arXiv.

[28] Novoselov K., Geim A.K., Morozov S.V., Jiang D., Katsnelson M.I., Grigorieva I.V., Dubonos S.V., Firsov A.A. (2005). Two-dimensional gas of massless Dirac fermions in graphene. Nature.

[29] Novoselov K., Jiang D., Schedin F., Booth T.J., Khotkevich V.V., Morozov S.V., Geim A.K. (2005). Two-dimensional atomic crystals. Proc. Natl. Acad. Sci. USA.

[30] Geim A.K., Grigorieva I.V. (2013). Van der Waals heterostructures. Nature.

[31] Lozovik Y.E., Sokolik A.A. (2008). Electron-hole pair condensation in a graphene bilayer. JETP Lett..

[32] Min H., Bistritzer R., Su J.-J., MacDonald A.H. (2008). Room-temperature superfluidity in graphene bilayers. Phys. Rev. B.

[33] Zhang C.-H., Joglekar Y.N. (2008). Excitonic condensation of massless fermions in graphene bilayers. Phys. Rev. B.

[34] Kharitonov M.Y., Efetov K.B. (2010). Excitonic condensation in a double-layer graphene system. Semicond. Sci. Technol..

[35] Lozovik Y.E., Ogarkov S.L., Sokolik A.A. (2010). Electron–electron and electron–hole pairing in graphene structures. Philos. Trans. R. Soc. A.

[36] Sodemann I., Pesin D.A., MacDonald A.H. (2012). Interaction-enhanced coherence between two-dimensional Dirac layers. Phys. Rev. B.

[37] Kac M., Murdock W.L., Szegö G. (1953). On the eigen-values of certain Hermitian forms. J. Rat. Mech. Anal..

[38] Rodman L., Shalom T. (1992). On inversion of symmetric Toeplitz matrices. SIAM J. Matrix Anal. Appl..

[39] Meurant G. (1992). Review on the Inverse of Symmetric Tridiagonal and Block Tridiagonal Matrices. SIAM J. Matrix Anal. Appl..

[40] Schutt M., Ostrovsky P.M., Titov M., Gornyi I.V., Narozhny B.N., Mirlin A.D. (2013). Coulomb Drag in Graphene Near the Dirac Point. Phys. Rev. Lett..

[41] Jia J., Gorbar E.V., Gusynin V.P. (2013). Gap generation in ABCstacked multilayer graphene: Screening versus band attening. Phys. Rev. B.

[42] Carrington M.E., Frey A.R., Meggison B.A. (2023). Effects of different 3D QED vertex Ansätze on the critical coupling. Phys. Rev. D.

[43] Gusynin V.P., Sharapov S.G., Carbotte J.P. (2007). AC conductivity of graphene: From tight-binding model to 2+1-dimensional quantum electrodynamics. Int. J. Mod. Phys. B.

[44] Elias D.C., Gorbachev R.V., Mayorov A.S., Morozov S.V., Zhukov A.A., Blake P., Ponomarenko L.A., Grigorieva I.V., Novoselov K.S., Guinea F. (2011). Dirac cones reshaped by interaction effects in suspended graphene. Nature Phys..

